# Hematological and Running Performance Modification of Trained Athletes after Reverse vs. Block Training Periodization

**DOI:** 10.3390/ijerph17134825

**Published:** 2020-07-04

**Authors:** Juan Pablo Gómez Martín, Vicente Javier Clemente-Suárez, Domingo Jesús Ramos-Campo

**Affiliations:** 1 Sport Science Faculty, Catholic University of Murcia, 30107 Murcia, Spain; jpgomezmartin0802@hotmail.com; 2 Universidad Europea de Madrid, Faculty of Sport Sciences, 28670 Villaviciosa de Odon, Spain; 3 Grupo de Investigación en Cultura, Educación y Sociedad, Universidad de la Costa, 080002 Barranquilla, Colombia; 4 Tritoledo Triathlon Club, 43560 Toledo, Spain

**Keywords:** endurance, heart rate, runners, triglycerides, VO*_2_*max

## Abstract

The aim of the present study was to analyze the effect of block (BP) and a reverse training periodization (RP) in the hematological and running performance of amateur trained athletes. Modifications in hematological, aerobic, and anaerobic running performance and countermovement jump before and after twelve weeks of BP vs. RP training programs were analyzed in 16 trained athletes (eight males: 40.0 ± 6.2 years; 179.2 ± 12.8 cm; 73.8 ± 12.2 kg; and eight females: 34.2 ± 4.1 years; 163.4 ± 9.6 cm; 57.0 ± 11.0 kg). A significant decrease in heart rate (HR) at ventilatory threshold (VT1) (*p* = 0.031; ES = 1.40) was observed in RP without changes in BP. In addition, RP increased significantly VO_2_max (*p* = 0.004; ES = 0.47), speed at VO_2_max (*p* = 0.001; ES = 1.07), HR at VT2 (*p* < 0.001; ES = 1.32) and VT1 (*p* = 0.046; ES = 0.57), while BP improved VO_2_max (*p* = 0.004; ES = 0.51), speed at VO*_2_*max (*p* = 0.016; ES = 0.92), and HR at VT2 (*p* = 0.023; ES = 0.78). In addition, only RP increased anaerobic performance in a running-based anaerobic sprint test (RAST) (mean sprint: *p* = 0.009; ES = 0.40, best sprint: *p* = 0.019; ES = 0.30 and total time: *p* = 0.009; ES = 0.40). Moreover, both types of training periodization proposed in this study maintained hematological values and efficiently improved jump performance (*p* = 0.044; ES = 0.6) in RP and *p* = 0.001; ES = 0.75 in BP). Therefore, twelve weeks of either RP or BP is an effective strategy to increase jump and aerobic running performance maintaining hematological values, but only RP increases anaerobic running performance.

## 1. Introduction

Sports performance is a complex combination of psychological and physiological modifications on the athlete’s organism. Specifically, in endurance athletes, the physiological modifications related with success have been clearly defined by previous researchers, in which maximal oxygen uptake (VO_2_max), lactate threshold, and efficiency appear to play key roles in endurance performance [1]. Along this line, the VO_2_max and lactate threshold interact to determine the maximal oxygen consumption that could be sustained for a given period of time, being the most used predictors of elite performance. As well as the anaerobic threshold, the ventilatory threshold is one of the physiological parameters related specifically with endurance and ultra-endurance performance, being a trainable parameter and having a direct effect on competition performance [2,3].

To reach the physiological adaptations previously mentioned, hematological and chemical modification must occur. Different modifications in red blood cells, blood iron, and iron reserve cells as well as in the metabolic substrate are dependent of the performance level reached by the athlete [4,5]. The continuous training and the rational distribution of the training sessions would be the pillars to obtain the correct physiological modifications in athletes. There are numerous training periodization models to reach this aim [6,7,8,9,10,11,12]: (i) the traditional periodization models, focused on long distance and low intensity training; (ii) block models, focused on concentrating training load in short time period to increase the organic adaptation, and (iii) the more recent training paradigm based on high intensity and low volume training called reverse periodization (RP).

RP, unlike previous periodization models, begin the macrocycle with high-intensity and low-volume training, while gradually decreasing intensity and increasing volume or, depending on the sport, maintaining intensity and increasing volume during the following training periods [13]. This recent training paradigm has been previously studied in physical fitness, strength training, swimming, triathlon and rowing, obtaining increases in muscular endurance, maximum strength, and endurance performance [10,13,14]. The increases in performance associated with the RP are closely related with the use of high intensity training, especially with methodology like high intensity interval training [7]. These short-term training methodologies have been reported as efficient interventions to increase sympathetic modulation to achieve different physiological adaptions related with aerobic performance such as the increase in muscle buffering capacity, glycogen content, GLUT4 concentration, and maximal glucose transport activity in skeletal muscle [15,16,17,18]. In addition, RP improves jump performance in endurance athletes, but traditional periodization affects it negatively [13].

Finally, hematological parameters might be influenced by long-term training and competition periods decreasing during the intense periods of training throughout the season [18]. However, the effect of periodized training, and specifically, the influence of this new training periodization model (i.e., reverse periodization) on chemical parameters and physiological adaptations are still poorly known, especially in the running collective. For this reason, we proposed the present research with the aim to analyze the effect of 12 weeks of a Block Periodization and a Reverse Periodization on hematological parameters, countermovement jump, and aerobic (measured in a treadmill test and a 10 km time trial test) and anaerobic running performance of trained athletes. The initial hypothesis was that reverse training periodization would achieve a higher running performance and a significant modification in hematological parameters than block training periodization.

## 2. Materials and Methods 

### 2.1. Design

To test the effects of 12 weeks of the two types of periodization training programs (reverse vs. block periodization) on hematological variables, aerobic and anaerobic running performance, and countermovement jump, a single-blinded randomized controlled (participant did not know the periodization model they were performing) trial with a pre- and post-test was conducted. Athletes were randomly divided into two experimental groups: (a) the reverse periodization (rp) group, who performed 4-weeks of high intensity training, 4-weeks of high volume training, and 4-weeks of tapering (*n* = 8); and (b) the block periodization (bp) group, who performed 4-weeks of high volume training (accumulation), 4-weeks of high intensity training (transformation), and 4-weeks of tapering (realization) (*n* = 8). 

### 2.2. Participants

Sixteen amateur athletes (eight males: 40.0 ± 6.2 years; 179.2 ± 12.8 cm; 73.8 ± 12.2 kg; and eight females: 34.2 ± 4.1 years; 163.4 ± 9.6 cm; 57.0 ± 11.0 kg; six training sessions/week; 42.4 ± 12.4 min/session; 4.3 ± 0.4 h of training/week; >6 year of experience on running training; competing at regional and national level in 10 km and half-marathon races) participated in this study. None of the participants had any musculoskeletal disorders. Before the testing sessions, participants were divided, in randomized order, into either the RP (*n* = 8; four males and four females; age: 37.0 ± 9.2 years; height: 170.2 ± 19.2 cm; weight: 65.8 ± 10.2 kg) or BP (*n* = 8; four males and four females; age: 37.2 ± 5.7 years; height: 172.4 ± 9.1 cm; weight: 65.1 ± 10.4 kg) groups. The study design and the procedures employed were in accordance with ethical standards and the Declaration of Helsinki (1964). Each participant was fully informed of the risks associated with the study and provided written informed consent before starting the study. The present research was approved by the Catholic University of Murcia Ethics Committee (REF: CE071902). 

### 2.3. Testing Protocol

The assessments were carried out on two different days, separated by 48 h, in both the pre- and post-test and at the same time of the day in both evaluations. Pre- and post-tests were carried out 72 h after the last intense workout to allow complete recovery from training. On the first day, athletes visited the laboratory to conduct a blood test, a running-based anaerobic sprint test (RAST), and a treadmill running test. The second testing session was performed 72 h after the first testing session on an official athletics track and included Countermovement Jump (CMJ) and the 10 km time trial test. On the day after the training program finished, the same testing procedures were applied. In addition, a nutritionist performed an initial prospective 24-h dietary recall to assess the participants’ diets. Afterward, a 7-day food record with qualitative and quantitative data, along with a printed guide for proper filling, was given to the participants to calculate their daily average intake through the first week of the training program, and calculated using software (Cronometer Software Inc., https://cronometer.com, V.1.) [19]. This nutritional assessment was performed again when the training program finished and no differences were observed between the periodization models and moments.

### 2.4. Blood Sample Collection

The blood sample (2.5 mL) was withdrawn from an antecubital vein using a sterile technique to analyze hematological variables. Blood samples were taken before breakfast after an overnight fast. Blood extraction was performed with the subject seated. Erythrocytes (×10^6^/L), hematocrit (%), hemoglobin (g/dL), ferritin (g/L), glucose (mg/dL), and triglycerides (mg/dL) were assessed.

### 2.5. Running-Based Anaerobic Sprint Test (RAST)

The RAST consisted of six maximal efforts of 35-m, separated by a passive recovery of 10 s. The athlete started 0.5 m behind the start line, which was marked by a photocell (Witty, Microgate, Italy) [20]. Before starting, the athletes were instructed to run as fast as possible to the end of the 35 m course. Before testing, a warm-up consisting of 5 min of jogging followed by active stretching and two short duration submaximal sprints was performed. Following each sprint, athletes decelerated and walked to the starting line ready for the subsequent sprint. The best and mean sprint time were recorded as the performance indices. Verbal encouragement was given to the participants to ensure maximum physical effort.

### 2.6. Treadmill Running Test

Thirty minutes after RAST test, runners completed an incremental test to exhaustion on a treadmill (Run MedTechnogym, Cessena, Italy) in standard environmental conditions with the grade set at 1%. The tests were performed between 10:30 and 12:00 a.m. in the laboratory with the room temperature set between 20°–22 °C and 45–50% of humidity. The gas analyzer system was calibrated before each test following the manufacturer’s recommendations. During testing, gas exchange was measured using a breath-by-breath gas analyzer (Metalyzer 3B; Cortex-medical, Leipzig, Germany). Expired minute volume (VE), oxygen consumption (VO_2_), and carbon dioxide production (VCO_2_) were continuously recorded and averaged each minute. The respiratory exchange ratio (R = VCO_2_/VO_2_), the O_2_ ventilatory equivalent (VE/VO_2_) and the CO_2_ ventilatory equivalent (VE/VCO_2_) were calculated. Athletes started running at 8 km/h for 5 min. Subsequently, the work rate was increased by 0.5 km/h every 30 s in a progressive manner until exhaustion for optimal determination of VT2 and VO_2_max. The corresponding heart rate was also determined by a validated Polar RS800CX heart rate monitor (Polar Electro, Kempele, Finland) [21]. Verbal encouragement was given to ensure maximum physical effort. The test was concluded according to traditional physiological criteria when participants reached volitional fatigue [3]. After the test, Ventilatory Thresholds were determined as follows: T1 was defined as the first increase of VE/VO_2_ vs. workload, without a simultaneous increase in VE/VCO_2_ vs. workload; and VT2 was defined as the second increase in VE with a concomitant rapid increase in VE/VO_2_ and VE/VCO_2_ and decrease of end-tidal CO_2_ tension (PETCO_2_) [22].

### 2.7. Countermovement Jump

During the second testing day, athletes completed a 15 min warm-up, similar to those performed prior to a competitive event, which included the following components: jogging (easy pace-Z1), running technique, and progressive running to race-pace. After warm-up, participants executed two submaximal trials of CMJ to ensure proper execution of the jumps with 1 min of rest in between trials. Two minutes after the specific warm-up to jump, participants started the CMJ test. For the CMJ execution, participants maintained 90° of knee flexion during 5″ while the researcher confirmed the 90° with a square. The CMJ heights were calculated using a contact platform (Ergotester, Globus, Codogne, Italy) [23]. The CMJ was performed at the center of the platform with the feet placed shoulder width apart in the standing position. Participants were asked to jump as high as possible with a rapid self-selected countermovement. Participants were asked to try and land close to the take-off point. Each participant performed two attempts, with 90 s of rest in between attempts. The best trial from each participant was used for data analysis.

### 2.8. Ten Kilometer Time Trial Test

After the CMJ test, a 10-km time trial test was carried out on an official athletic track. The 10-km time was recorded using a Geonaute chronometer Onstart 710 (Decathlon, Villeneuve-d’Ascq, France) by two of the researchers and the mean of these values was used for analysis.

### 2.9. Training Program

Two weeks before starting the training program, all participants performed the same two-familiarization weeks. During this stage, athletes completed three running sessions in Z1 and two strength workouts each week. Participants started the familiarization period after three weeks of detraining weeks or off-season period. After this initial training period, participants in both periodizations completed a 12-week training periodization program consisting of two strength workouts, five running session, and one day of total rest per week. The RP group performed a 12-week periodization composed of a 4-week mesocycle based on high intensity and low volume training, a 4-week mesocycle based on high volume and intensity training, and a 4-week mesocycle based on modeling competition and tapering. The training intensity distribution was polarized in the first mesocycle and pyramidal in the second and third. On the other hand, the BP group completed a 12-week periodization composed of 4-weeks of high volume training (accumulation), 4-weeks of high intensity training (transmutation), and 4-weeks of modeling competition and tapering (realization). In addition, we applied a polarized distribution in the second mesocycle and a pyramidal distribution in the first and third. Training zones were classified, according to previous literature, in three training zones: zone 1 (Z1), low intensity training (Rated of Perceived Exertion, RPE ≤ 4); zone 2 (Z2), anaerobic threshold training (RPE 4–7); and zone 3 (Z3), high intensity training (RPE ≥ 7) [24]. To quantify the training load of each session conducted by the athletes during each week, we used the session-RPE method [25]. In this method, the training load is quantified by multiplying the whole training-session RPE using the 10-point Borg scale by its duration. This product represents the training impulse (TRIMP) or the magnitude of the internal training load in arbitrary units. The RPE was recorded thirty minutes after every training session. An example of typical training series in each phase of the 12-week training period for both periodizations are shown in Table 1.

### 2.10. Statistical Analysis

Statistical analysis of data was performed with SPSS v 24.0 (Chicago, IL, USA) in the Windows environment. Descriptive data are presented as mean ± standard deviation (SD). For the inferential analysis, a Shapiro–Wilks W-Test was performed to establish the normality of the sampling distribution and Mauchly’s W test to analyze the sphericity between measurements. In addition, a two-way (type of periodization × time) analysis of variance (ANOVA) with repeated measures and Bonferroni post-hoc was used to investigate the differences in the study variables. In addition, an unpaired sample *T*-test was used to compare the training load of both periodization programs. Mean difference and 95% confidence interval (95% CI) were included. The effect size (ES) was calculated using partial ETA squared (η2) in ANOVA. In addition, the d was calculated using Cohen’s guidelines to compare the training load in each intervention (BP vs. RP) and to analyze the effect of time using the threshold values of >0.2 (small), >0.6 (moderate), >1.2 (large), and >2.0 (very large) [26]. For all procedures, a level of *p* ≤ 0.05 was selected to indicate statistical significance.

## 3. Results

Table 2 shows the hematological variables. There was no interaction effect of periodization group × time in the hematological variables. There was a main effect of time on triglycerides (*F* = 5.333; *p* = 0.037; d = 0.94) in RP.

Concerning the running treadmill test variables investigated (Table 3), a significant interaction effect of periodization × time was observed in the heart rate at VT1 (*F* = 7.11; *p* = 0.018; η^2^ = 0.34), showing a significant decrease in RP (*F* = 4.74; *p* = 0.031; d = 1.40) but without changes in BP. In addition, there was a main effect of time on HR at VT2 (*F* = 26.57; *p* < 0.001; d = 1.32), VT1 (*F* = 5.74; *p* = 0.046; d = 0.57), VO*_2_*max (*F* = 11.93; *p* = 0.004; d = 0.47), and speed of VO_2_max (*F* = 15.73; *p* = 0.001; d = 1.07) in RP and on HR at VT2 (*F* = 5.49; *p* = 0.023; d = 0.78), VO_2_max (*F* = 11.93; *p* = 0.004; d = 0.51), and speed of VO_2_max (*F* = 7.56; *p*= 0.016; d = 0.92) in BP.

Regarding the RAST variables (Table 4), there was a main effect of group × time on mean time in RAST (s) (*F* = 9.32; *p* = 0.009; η^2^ = 0.40) and on the total time of the RAST test (s) (*F* = 9.32; *p* = 0.009; η^2^ = 0.40) with a significant decrease in RP. Furthermore, there was a main effect of time on the best sprint in RAST (s) (*F* = 7.09; *p* = 0.019; d = 0.30) in RP. Concerning CMJ and the 10,000 m test variables analyzed (Table 3), no main effect of periodization group × time was observed in these variables. However, there was a main effect of time on CMJ height (*F* = 4.54; *p* = 0.044; d = 0.6) in RP and in CMJ height (*F* = 14.72; *p*= 0.001; d = 0.75) and the 10 km time-trial (*F* = 9.73; *p* = 0.008; d = 0.15) in BP.

**Figure 1:**
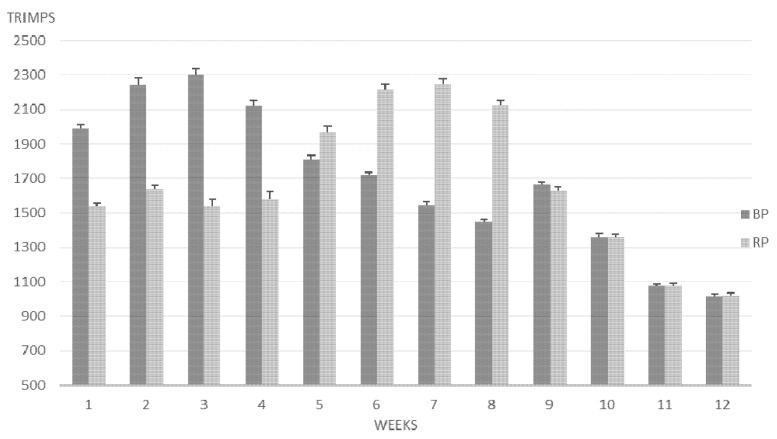
Training load (TRIMPS) in arbitrary units during the 12 weeks of the two periodization models (RP: reverse periodization; BP: block periodization).

Concerning training monitoring, significant differences were observed in the total training load (TRIMPS), total training time (min), and in the time spent in each training zone between groups (Table 5 and Figure 1).

## 4. Discussion

The aim of this study was to analyze the effect of BP vs. RP in the hematological and running performance of amateur trained athletes. The initial hypothesis was partially confirmed since RP achieved an increased running performance as well as BP, with only triglycerides modified by RP. On the other hand, RP achieved higher anaerobic running performance than BP.

Analyzing the training load of each group, we found a significant higher volume in BP than RP. Traditional periodization studies tried to equal the volume of training in the different training groups analyzed, producing a distortion in the reverse training periodization structure in this matching process [13]. Reverse periodization is based on a low training volume combined with high intensity, starting the implementation of high intensity from the beginning of the macrocycle [23]. Previous authors have shown how the equalization of training volumes and intensities when comparing reverse periodization with other models was a limitation to really testing the efficiency of this new training paradigm [6,27,28]. In the present study, we took that fact into account, and the load was distributed according to this premise, obtaining a significant difference in training volumes.

The reverse periodization showed an increase in cardiovascular efficiency in both VT1 and VT2. This modification could be due to the effect of high-intensity training conducted in this periodization model, which led to a hyperactivation of the sympathetic nervous system during the efforts, being a powerful tool to lead this cardiovascular improvement [7,18]. The increases in VO_2_max and the speed at VO_2_max obtained by reverse and BP highlighted the effectiveness of both systems to reach aerobic physiological markers, but reverse periodization was more efficient since a lower training volume was performed. The increases in VO_2_max and speed at VO_2_max were in consonance with previous authors in equaled volume training periodization in cycling, triathlon, and swimming [12,13,23], showing how these methods also improve aerobic performance in running. In the same way, the decreased in HR at VT2 was in line with other research conducted with swimmers [6]; nevertheless, in this study, improvement in swimming speed was found, in contrast to the present research, where most likely the different performance level and sport modality could explain this difference.

Both training groups (RP and BP) significantly increased the VO_2_max, but only in BP was this increase accompanied by a significant improvement in the running field test (10,000 m).This result was in contrast with previous research where after 10 weeks of reverse periodization, running performance (400 m and 1000 m) increased in amateur athletes [29]. We found how reverse periodization allowed for an increase in physiological laboratory test markers, but in this case failed to increase field performance. This result should take into account for a better design of this specific periodization, trying to improve the transference of training adaptations to final field performance. In this line, the significative improvements of block periodization could be related with the higher volume of training in the Z2 and Z3 intensity zones, specific to improving performance in the field test analyzed [12].

Regarding jump performance results, a previous study reported an interference effect of aerobic endurance exercise on strength and power gains [12]. In addition, a recent study showed that traditional periodization negatively affected jump performance in endurance athletes, but reverse periodization improved it [13]. In this way, our results showed that variations in training periodization could interfere with lower body power in both groups, suggesting that both RP and BP positively improved CMJ height. This fact could be explained by the lower load performed by the two groups during the last weeks of the program (tapering), which is in accordance with a previous study that found an increase in jump performance during the last two weeks of a training program where the load decreased in endurance athletes [30]. Therefore, both RP and BP are effective periodization programs to improve CMJ jump in amateur athletes.

Concerning hematological parameters, hemoglobin and hematocrit might be influenced by long-term training and competition periods decreasing during the intense periods of training throughout the season [31]. According to our results, the type of periodization does not seem to affect the hematological variables analyzed in this study, showing that the two types of periodization reported a non-significant trend to decrease. This fact could be explained by the production of a hemolysis or/and hemodilution, which can contribute to generating sport anemia [32], which in a clinical case can impair athletic performance [33]. Thus, both training periodization of RP focusing more in Z3 and BP with more total training load and more time training in Z1 and Z2 can produce a decreasing trend in hematological parameters. In addition, our results showed a significant increase in blood triglycerides in RP but not in BP. Despite the values of triglycerides in both cases being in a physiological range, this fact could be explained by the training distribution performed in RP. The high-intensity training (Z3) increased post-exercise lipid oxidation, decreased triglycerides levels [34], and a higher amount of training of this type of exercise is performed at the beginning of the RP. In addition, triglycerides and other lipid metabolism parameters are strongly dependent on the training level of athletes, impairing during short-term detraining periods [35]. Therefore, more studies are needed to explain the long-terms effect of RP in fat metabolism and the response of other variables like body composition to this type of periodization.

A previous study showed that RP periodization can optimize anaerobic running performance (i.e., 400 m running) in comparison to BP [29]. We found similar results in the present research, showing that RP, but not BP, significantly improved RAST performance. This fact could be explained by the higher time training performed in Z3 of RP than the BP. The training performed in this zone (i.e., high-intensity interval training) improved anaerobic pathways, muscle buffering capacity, and lactate tolerance [15,35,36], obtaining higher anaerobic performance. Therefore, RP could be a good strategy to improve anaerobic performance in amateur endurance athletes in order to obtain a successful performance in running races where runners sometimes need to perform a sprint at the end of the race, but future research with a larger sample should confirm this point.

The main limitation of the present study was the small sample size analyzed, which limited the generalization of the results. Along this line, we could not perform a randomized controlled crossover because of the impossibility of maintaining participants during a long time of period out of their club training and competitions. In addition, regarding training volumes, we wanted to analyze the real training proposed by coaches following both periodization models, and not only the concept of reverse periodization. Then, a reduced volume was proposed for the reverse training periodization once it was one of the bases of this periodization model. As a consequence, our conclusions should not be extracted regarding only the intense training distribution during the macrocycle, but to the cumulative effect of this with the usual reduction in overall training volume, which is the paradigm of the reverse training periodization: low volume and high intensity since the start of the macrocycle.

In addition, practical recommendations should be restricted to amateur runners. Nevertheless, due to the findings in aerobic performance, it can be reasonably suggested from our data that the findings of this study can apply to other athletes such as endurance athletes who may want to optimize their training program. However, more research with endurance athletes is necessary to obtain more information about the most effective periodization in populations and athletes with different fitness levels. Furthermore, from an applied perspective, the athletes’ coaches and research have information to help them in training planification. They can use the results obtained to select the most effective periodization according to the physical demands of the sport modality, given that RP improved both aerobic and anaerobic performance. This periodization, focused on intensity, led us to think that intensified training is a key factor in optimizing endurance athlete performance.

The basis of reverse training periodization is a decrease in low intensity-high volume training and an increase in high intensity-low volume training. As a future line of research, we propose an analysis to address the influence of each individual factor in the final performance of athletes to better understand the principles of this new training paradigm.

## 5. Conclusions

Reverse and block periodization are an effective strategy to improve physiological variables and aerobic running performance during a treadmill test, but only reverse periodization increased anaerobic running performance in a RAST test. Moreover, both types of training periodization proposed in this study maintained the hematological values of the amateur athletes. In addition, ten weeks of a reverse periodization program increased blood triglyceride values. Moreover, both types of periodization efficiently improved jump performance.

## Figures and Tables

**Table 1:** Examples of typical training series in each phase of the 12-week training period for both block and reverse training periodizations.

Periodization Model	Weeks 1–4	Weeks 5–8	Weeks 9–12
Block periodization	1 × 50 min Z1 3 × 2000 m/3 min Z2	6 × 1000 m/3 min Z3 2 × (5 × 400 m/90 s)/8 min/Z3	10 × 1000 m/3 min Z3
Reverse periodization	10 × 200 m/2 min Z3 3 × (10 × 100 m/30 s)/3′ Z3	8 × 1000 m/2 min Z2 2 × (10 × 300 m/90 s)/8 min Z3	10 × 1000 m/3 min Z3

Series × (repetition × distance or duration and intensity/recovery between repetitions)/recovery between series; Z1—Low intensity training; Z2—Threshold training; Z3—High intensity training.

**Table 2:** Hematological results in both groups.

Variables	Pre-Training		Post-Training				95% CI for Difference		
	Mean	SD	Mean	SD	ES	*p*	MD	Lower Bound	Upper Bound
Reverse Periodization									
Erythrocytes (×10^6^/L)	4.5	0.1	4.4	0.1	0.98	0.299	−0.1	−0.3	0.1
Hematocrit (%)	42.9	1.1	42.4	0.8	0.37	0.448	−0.5	−1.8	0.8
Hemoglobin (g/dL)	14.2	0.4	13.8	0.2	0.81	0.129	−0.4	−0.9	0.1
Ferritin (g/L)	129.8	31.8	124.1	19.4	0.16	0.627	−5.7	−30.1	18.5
Glucose (mg/dL)	88.7	3.2	84.7	1.8	1.1	0.077	−4	−8.4	0.4
Triglycerides (mg/dL)	70	5.4	75.7	4.5	−0.94	0.037	5.7	1.1	10.3
Block Periodization									
Erythrocytes (×10^6^/L)	4.5	0	4.5	0	0.89	0.164	−0.1	−0.2	0.1
Hematocrit (%)	42.1	0.9	41	0.6	0.98	0.107	−1.1	−2.4	0.2
Hemoglobin (g/dL)	14.1	0.3	13.6	0.2	1.55	0.1	−0.5	−1	0
Ferritin (g/L)	118.5	19.7	108.3	14.7	0.46	0.396	−10.2	−34.5	14.2
Glucose (mg/dL)	90.7	3.2	87.7	1.8	0.83	0.177	−3.1	−7.4	1.4
Triglycerides (mg/dL)	82.7	5.6	83.6	4.3	−0.14	0.698	0.9	−3.7	5.5

MD: Mean difference. CI: Confident interval; ES: effect size.

**Table 3:** Treadmill running test results in both groups.

Variables	Pre-Training	SD	Post-Training		ES	*p*	95% CI for Difference		
	Mean		Mean	SD			MD	Lower Bound	Upper Bound
Reverse Periodization									
HR VT1 (bpm)	140.3	2.0	137.1	2.5	1.40	0.031	−3.3	−6.1	−0.3
Speed VT1 (km/h)	10.8	1.0	11.2	0.9	−0.35	0.278	0.3	−0.3	1
VT1 (% of VO_2_max)	59.9	1.6	60.9	1.6	−0.57	0.046	1	0	1.9
HR VT2 (bpm)	177.0	2.8	172.9	2.4	1.32	<0.001	−4.1	−5.7	−2.7
Speed VT2 (km/h)	15.4	0.8	15.7	0.9	−0.27	0.322	0.3	−0.2	0.7
VT2 (% of VO_2_max)	71.3	1.4	71.6	1.5	−0.24	0.548	0.4	−0.9	1.6
HR VO_2_max (bpm)	187.6	1.7	187.8	1.8	−0.07	0.661	0.1	−0.4	0.7
Speed VO_2_max (km/h)	17.5	0.7	18.3	0.6	−1.07	<0.001	0.8	−2.3	−1.2
VO_2_max (ml/kg/min)	59.4	2.6	60.8	2.6	−0.47	0.004	1.4	0.5	2.1
Block Periodization									
HR VT1 (bpm)	138.8	2.5	141.2	1.8	−0.83	0.106	2.4	−0.5	5.3
Speed VT1 (km/h)	11.3	0.5	11.2	0.9	0.13	0.364	−0.1	−1	0.3
VT1 (% of VO_2_max)	62.6	1.4	62.9	1.2	−0.16	0.602	0.3	−0.7	1.2
HR VT2 (bpm)	170.8	2.1	168.9	1.7	0.78	0.023	−1.9	−3.4	−0.2
Speed VT2 (km/h)	14.4	0.5	14.9	0.6	−0.91	0.056	0.5	0	1
VT2 (% of VO_2_max)	72.5	2.1	72.0	1.6	0.2	0.448	−0.4	−1.7	0.8
HR VO_2_max (bpm)	183.8	1.6	183.9	1.7	−0.07	0.661	0.1	−0.4	0.7
Speed VO_2_max (km/h)	16.8	0.5	17.4	0.6	−0.92	0.016	0.6	−2.5	−1.3
VO_2_max (ml/kg/min)	53.6	2.4	55.0	2.1	−0.51	0.004	1.4	0.5	2.1

HR: heart rate; VT: ventilatory threshold; VO_2_max: maximum oxygen uptake; MD: mean difference; CI: confident interval; ES: effect size.

**Table 4:** Countermovement jump test, RAST test, and 10,000 m time trial test results in both groups.

Variables	Pre-Training		Post-Training				95% CI for Difference		
	Mean	SD	Mean	SD	ES	*p*	MD	Lower Bound	Upper Bound
Reverse Periodization									
Mean sprint RAST (s)	5.8	0.3	5.6	0.3	0.59	0.01	−0.2	−0.1	−0.3
Best sprint RAST (s)	5.6	0.3	5.5	0.3	0.3	0.019	−0.1	−0.2	0
Total time sprint RAST (s)	34.8	1.9	33.6	1.8	0.56	0.01	−1.2	−0.6	−1.8
CMJ height (cm)	33	1.5	34	1.2	−0.6	0.044	1	0	1.9
10,000 m (s)	2481.5	369.4	2429.2	363.6	0.13	0.089	−52	−113	9
Block Periodization									
Mean sprint RAST (s)	5.7	0.4	5.7	0.4	0	0.965	0	0.1	−0.1
Best sprint RAST (s)	5.6	0.4	5.6	0.4	0	1	0	0.1	−0.1
Total time sprint RAST (s)	34.2	2.7	34.2	2.4	0	0.965	0	0.6	−0.6
CMJ height (cm)	31.3	2.1	33.1	1.8	−0.75	0.001	1.8	0.8	2.7
10,000 m (s)	2728.8	522.3	2640	418.3	0.15	0.008	−88.8	−149.8	−27.7

RAST: running-based anaerobic sprint test; CMJ: countermovement jump; MD: mean difference; CI: confident interval; ES: effect size.

**Table 5:** Training load of each periodization group.

	Total Time (min)	Time in Z1 (min)	Time in Z1 (%)	Time in Z2 (min)	Time in Z2 (%)	Time in Z3 (min)	Time in Z3 (%)	Training Load (TRIMPS)
Reverse Periodization	3246.1 ± 38.3	1963.0 ± 30.0	60.5 ± 0.5	715.6 ± 6.1	22.5 ± 0.3	567.6 ± 20.4	17.5 ± 0.5	19,932.1 ± 250.5
Block Periodization	3319.9 ± 37.4	2009.4 ± 25.3	60.5 ± 0.2	774.4 ± 11.5	23.3 ± 0.2	536.1 ± 4.7	16.2 ± 0.1	20,292.3 ± 222.0
*p*	0.002	0.005	0.780	<0.001	<0.001	0.001	<0.001	0.009
ES (*d*)	1.95	1.58	0	6.04	2.97	2.01	3.41	1.52

Zone 1 (Z1), low intensity training; zone 2 (Z2), anaerobic threshold training; and zone 3 (Z3) high intensity training.
